# Supplementary material for: OpenMM 7: Rapid development of high performance algorithms for molecular dynamics
Source: PLoS Comput Biol. 2017 Jul 26;13(7):e1005659. doi: 10.1371/journal.pcbi.1005659 (PMC5549999; doi:10.1371/journal.pcbi.1005659)
Supplement: S1 Source Code — (ZIP) [file pcbi.1005659.s002.zip › OpenMM-7.0.1-Source/docs/api-python/generated/simtk.openmm.openmm.AmoebaTorsionTorsionForce.html]

AmoebaTorsionTorsionForce — OpenMM 7.0.1.dev-5e86c4f documentation


# AmoebaTorsionTorsionForce¶

*class* `simtk.openmm.openmm.``AmoebaTorsionTorsionForce`(*\*args*)¶
:   This class implements the Amoeba torsion-torsion interaction.

    To use it, create an AmoebaTorsionTorsionForce object then call addTorsionTorsion() once for each torsion-torsion. After a torsion-torsion has been added, you can modify its force field parameters by calling setTorsionTorsionParameters().

    `__init__`(*self*) → AmoebaTorsionTorsionForce¶
    :   \_\_init\_\_(self, other) -> AmoebaTorsionTorsionForce

        Create an AmoebaTorsionTorsionForce.

    Methods

    |  |  |
    | --- | --- |
    | `__init__`((self) -> AmoebaTorsionTorsionForce) | \_\_init\_\_(self, other) -> AmoebaTorsionTorsionForce |
    | `addTorsionTorsion`((self, particle1, ...) | Add a torsion-torsion term to the force field. |
    | `getForceGroup`((self) -> int) | Get the force group this Force belongs to. |
    | `getNumTorsionTorsionGrids`((self) -> int) | Get the number of torsion-torsion grids |
    | `getNumTorsionTorsions`((self) -> int) | Get the number of torsion-torsion terms in the potential function |
    | `getTorsionTorsionGrid`((self, index) -> vectorddd) | Get the torsion-torsion grid at the specified index |
    | `getTorsionTorsionParameters`(self, index) | Get the force field parameters for a torsion-torsion term. |
    | `setForceGroup`(self, group) | Set the force group this Force belongs to. |
    | `setTorsionTorsionGrid`(self, index, grid) | Set the torsion-torsion grid at the specified index |
    | `setTorsionTorsionParameters`(self, index, ...) | Set the force field parameters for a torsion-torsion term. |
    | `usesPeriodicBoundaryConditions`((self) -> bool) | Returns whether or not this force makes use of periodic boundary conditions. |

    `getNumTorsionTorsions`(*self*) → int¶
    :   Get the number of torsion-torsion terms in the potential function

    `getNumTorsionTorsionGrids`(*self*) → int¶
    :   Get the number of torsion-torsion grids

    `addTorsionTorsion`(*self*, *particle1*, *particle2*, *particle3*, *particle4*, *particle5*, *chiralCheckAtomIndex*, *gridIndex*) → int¶
    :   Add a torsion-torsion term to the force field.

        |  |  |
        | --- | --- |
        | Parameters: | - **particle1** (*int*) – the index of the first particle connected by the torsion-torsion - **particle2** (*int*) – the index of the second particle connected by the torsion-torsion - **particle3** (*int*) – the index of the third particle connected by the torsion-torsion - **particle4** (*int*) – the index of the fourth particle connected by the torsion-torsion - **particle5** (*int*) – the index of the fifth particle connected by the torsion-torsion - **chiralCheckAtomIndex** (*int*) – the index of the particle connected to particle3, but not particle2 or particle4 to be used in chirality check - **gridIndex** (*int*) – the index to the grid to be used |
        | Returns: | the index of the torsion-torsion that was added |
        | Return type: | int |

    `getTorsionTorsionParameters`(*self*, *index*)¶
    :   Get the force field parameters for a torsion-torsion term.

        |  |  |
        | --- | --- |
        | Parameters: | **index** (*int*) – the index of the torsion-torsion for which to get parameters |
        | Returns: | - **particle1** (*int*) –   the index of the first particle connected by the torsion-torsion - **particle2** (*int*) –   the index of the second particle connected by the torsion-torsion - **particle3** (*int*) –   the index of the third particle connected by the torsion-torsion - **particle4** (*int*) –   the index of the fourth particle connected by the torsion-torsion - **particle5** (*int*) –   the index of the fifth particle connected by the torsion-torsion - **chiralCheckAtomIndex** (*int*) –   the index of the particle connected to particle3, but not particle2 or particle4 to be used in chirality check - **gridIndex** (*int*) –   the grid index |

    `setTorsionTorsionParameters`(*self*, *index*, *particle1*, *particle2*, *particle3*, *particle4*, *particle5*, *chiralCheckAtomIndex*, *gridIndex*)¶
    :   Set the force field parameters for a torsion-torsion term.

        |  |  |
        | --- | --- |
        | Parameters: | - **index** (*int*) – the index of the torsion-torsion for which to set parameters - **particle1** (*int*) – the index of the first particle connected by the torsion-torsion - **particle2** (*int*) – the index of the second particle connected by the torsion-torsion - **particle3** (*int*) – the index of the third particle connected by the torsion-torsion - **particle4** (*int*) – the index of the fourth particle connected by the torsion-torsion - **particle5** (*int*) – the index of the fifth particle connected by the torsion-torsion - **chiralCheckAtomIndex** (*int*) – the index of the particle connected to particle3, but not particle2 or particle4 to be used in chirality check - **gridIndex** (*int*) – the grid index |

    `getTorsionTorsionGrid`(*self*, *index*) → vectorddd¶
    :   Get the torsion-torsion grid at the specified index

        |  |  |
        | --- | --- |
        | Parameters: | **index** (*int*) – the grid index |
        | Returns: | **vector< std** – grid return grid reference |
        | Return type: | :vector< std::vector< double > > > |

    `setTorsionTorsionGrid`(*self*, *index*, *grid*)¶
    :   Set the torsion-torsion grid at the specified index

        |  |  |
        | --- | --- |
        | Parameters: | - **index** (*int*) – the index of the torsion-torsion for which to get parameters - **grid** (*vector< std::vector< std::vector< double > > >*) – either 3 or 6 values may be specified per grid point. If the derivatives are omitted, they are calculated automatically by fitting a 2D spline to the energies. grid[x][y][0] = x value grid[x][y][1] = y value grid[x][y][2] = energy grid[x][y][3] = dEdx value grid[x][y][4] = dEdy value grid[x][y][5] = dEd(xy) value |

    `usesPeriodicBoundaryConditions`(*self*) → bool¶
    :   Returns whether or not this force makes use of periodic boundary conditions.

        |  |  |
        | --- | --- |
        | Returns: | true if nonbondedMethod uses PBC and false otherwise |
        | Return type: | bool |

    `__delattr__`¶
    :   x.\_\_delattr\_\_(‘name’) <==> del x.name

    `__format__`()¶
    :   default object formatter

    `__getattribute__`¶
    :   x.\_\_getattribute\_\_(‘name’) <==> x.name

    `__hash__`¶

    `__reduce__`()¶
    :   helper for pickle

    `__reduce_ex__`()¶
    :   helper for pickle

    `__sizeof__`() → int¶
    :   size of object in memory, in bytes

    `__str__`¶

    `getForceGroup`(*self*) → int¶
    :   Get the force group this Force belongs to.

    `setForceGroup`(*self*, *group*)¶
    :   Set the force group this Force belongs to.

        |  |  |
        | --- | --- |
        | Parameters: | **group** (*int*) – the group index. Legal values are between 0 and 31 (inclusive). |

High performance molecular simulation on GPUs


### Navigation

- Application Layer
- Library Layer
  - Core Objects
  - Forces
  - Integrators
  - Extras

©2015, Stanford University and the Authors.
|
Powered by Sphinx 1.3.1
& Alabaster 0.7.7
|
Page source
